# Supplementary material for: Formulation, Colloidal Characterization, and In Vitro Biological Effect of BMP-2 Loaded PLGA Nanoparticles for Bone Regeneration
Source: Pharmaceutics. 2019 Aug 3;11(8):388. doi: 10.3390/pharmaceutics11080388 (PMC6723283; doi:10.3390/pharmaceutics11080388)
Supplement: Supplementary file 1 [file pharmaceutics-11-00388-s001.zip › pharmaceutics-542449-sm online/supplementary materials - figure S 1 2.docx]

Supplementary Material: Formulation, Colloidal Characterization, and In Vitro Biological Effect of BMP-2 Loaded PLGA Nanoparticles for Bone Regeneration

Teresa del Castillo-Santaella ^1^ , Inmaculada Ortega-Oller ^2,^ , Miguel Padial-Molina ^2^, Francisco O’Valle^3^, Pablo Galindo-Moreno ^2^, Ana Belén Jódar-Reyes ^1,4^ and José Manuel Peula-García ^1,5,^*


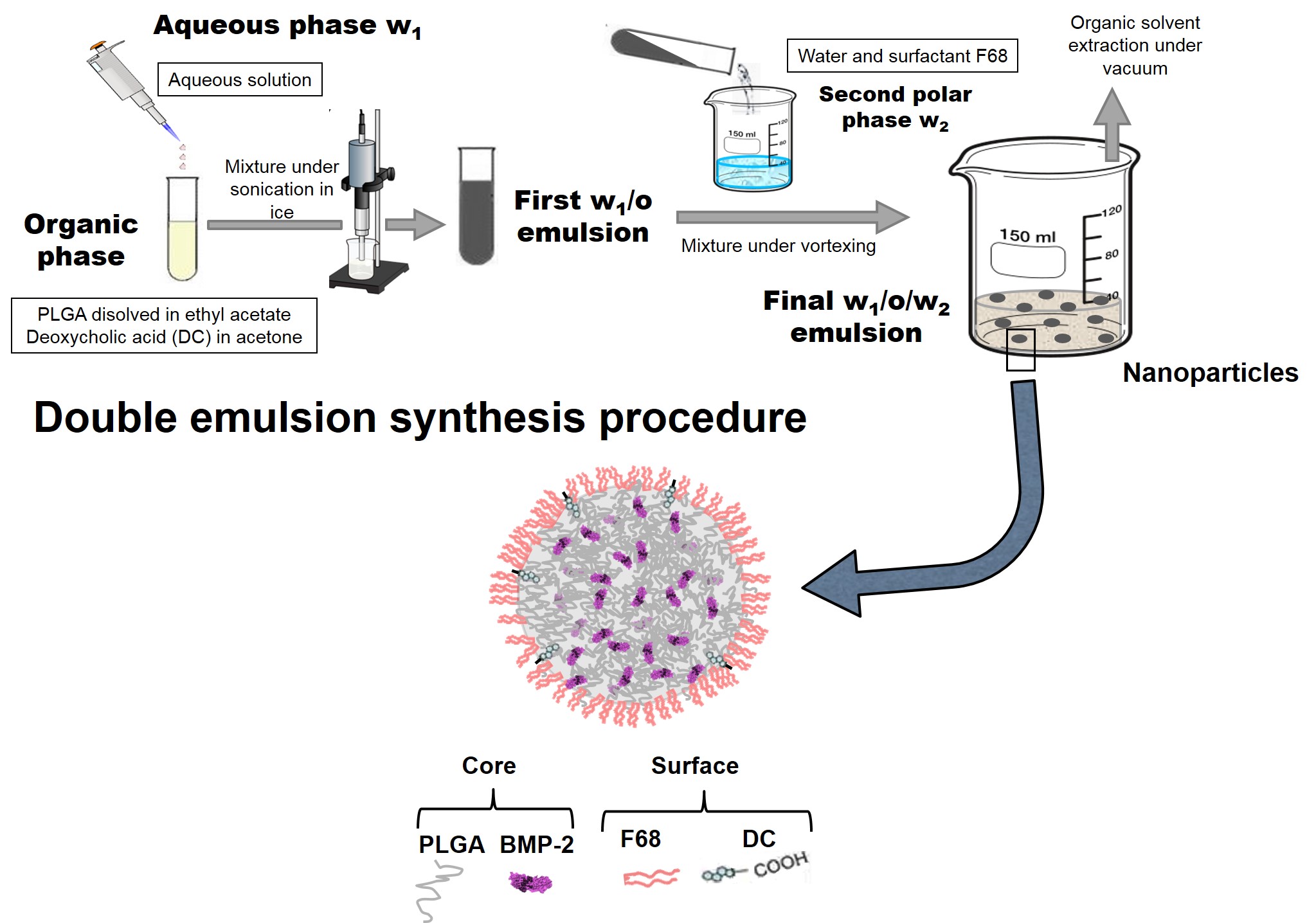


**Figure 1.** Scheme of the formulation of NP-BMP2 [1].


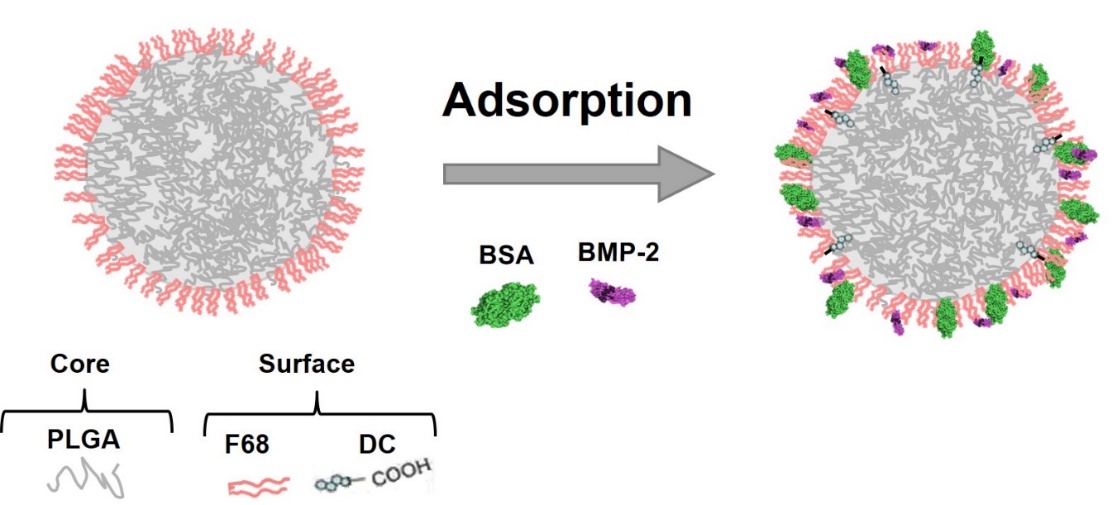


**Figure 2.** Scheme of the protein adsorption process for NP-BSA-BMP2.

Reference

1. Ortega-Oller, I.; del Castillo-Santaella, T.; Padial-Molina, M.; Galindo-Moreno, P.; Jódar-Reyes, A.B.; Peula-García, J.M. Dual delivery nanosystem for biomolecules. Formulation, characterization, and in vitro release. *Coll. Surf. B Biointerface.* **2017**, *159*, 586–595, doi:10.1016/j.colsurfb.2017.08.027.
